# Assessment of Environmental and Hereditary Influence on Development of Pituitary Tumors Using Dermatoglyphic Traits and Their Potential as Screening Markers 

**DOI:** 10.3390/ijerph13030330

**Published:** 2016-03-17

**Authors:** Marina Gradiser, Martina Matovinovic Osvatic, Dario Dilber, Ines Bilic-Curcic

**Affiliations:** 1 County Hospital Cakovec, I.G. Kovacica 1E, 40000 Cakovec, Croatia; marina.gradiser@gmail.com (M.G.); dario.dilber@gmail.com (D.D.); 2 Department of Endocrinology, University Hospital Centre Zagreb, Kišpatićeva 12, 10000 Zagreb, Croatia; martina1000@yahoo.com; 3 Faculty of Medicine, University Josip JurajStrossmayer, Department of Pharmacology, Clinical Hospital Center Osijek, 31000 Osijek, Croatia

**Keywords:** dermatoglyphic traits, functional pituitary tumors, non-functional pituitary tumors, early detection, multifactorial etiology

## Abstract

The aim of this study was to assess environmental and hereditary influence on development of pituitary tumors using dermatoglyphic traits. The study was performed on 126 patients of both genders with pituitary tumors (60 non-functional and 66 functional pituitary tumor patients) in comparison to the control group of 400 phenotypically healthy individuals. Statistical analysis of quantitative and qualitative traits of digito-palmar dermatoglyphics was performed, and hormonal status was determined according to the standard protocols. Although we did not find markers that could specifically distinguish functional from non-functional tumors, we have found markers predisposing to the development of tumors in general (a small number of ridges between triradius of both hands, a smaller number of ridges between the triradius of c–d rc R), those for endocrine dysfunction (increased number of arches and reduced number of whorls, difference of pattern distribution in the I3 and I4 interdigital space), and some that could potentially be attributed to patients suffering from pituitary tumors (small number of ridges for variables FRR 5, smaller number of ridges in the FRL 4 of both hands and difference of pattern distribution at thenar of I1 and I2 interdigital space). The usage of dermatoglyphic traits as markers of predisposition of pituitary tumor development could facilitate the earlier detection of patients in addition to standard methods, and possibly earlier treatment and higher survival rate. Finally, our results are consistent with the hypothesis about multifactorial nature of pituitary tumor etiology comprised of both gene instability and environmental factors.

## 1. Introduction

Pituitary adenomas are the most frequent pituitary tumors, detrimental because of their localization. They result in clinical sequelae and accelerated mortality due to central mass effects or pituitary hormone hypersecretion and/or insufficiency [1]. Their diameter is generally less than 10 mm (microadenomas), but sometimes could be 10 mm to 5 cm or larger (macroadenomas), in which case they are usually non-functional (about 20% of all pituitary tumors). Clinical presence depends on their localization and hormonal activity. As indicated by Molitch [2], silent pituitary microadenomas are identified in approximately 11% of autopsy specimens. 

Most pituitary tumors are pituitary adenomas, benign, slow growing tumors which can be secretory or non functional (endocrine inactive) according to their ability to produce hormones. Symptoms of an adenoma are unspecific and depend on their size and secreting activity thus delaying timely diagnosis of such lesions [3,4,5,6]. Most commonly they include headaches, vision problems, menstrual cycle abnormality, erectile dysfunction or weight change. There are few risk factors for pituitary tumors and these are all related to genetics [7,8], but there are no known environmental or lifestyle-related risk factors for pituitary tumors.

Dermatoglyphics are patterns observed on the epidermis on the fingers, palms, and soles. They are completely formed by week 21 of intrauterine development and, furthermore, totally resistant to any external factor, remaining unchanged until the end of a person’s life and hence used for personal identification [9]. Analysis of dermatoglyphic traits is accepted as a simple and inexpensive method for deciding whether a patient has a particular genetic disorder or not. Therefore, studying dermatoglyphics contributes to our better understanding of genetic status and early intrauterine development, which makes them applicable in biomedical sciences [10].

Previous findings have shown the correlation between some properties of digito-palmary dermatoglyphics and certain tumors [11,12,13]. Therefore, except for the possibility of pointing to a genetic/epigenetic cause of a disease, dermatoglyphics could also serve as a high-quality, easily available, and cheap marker for the discrimination of the group with a predisposition for developing certain diseases [11,12,13,14,15,16]. The aim of this study was to assess environmental and hereditary influences on the development of pituitary tumors using dermatoglyphic traits and to determine whether there is a distinctive pattern of dermatoglyphics in patients with those tumors. If a meaningful association can be established, dermatoglyphics could be used for inexpensive and non-invasive screening of populations at risk leading to anticipation and early detection of symptoms, which could help in averting the disease or complications associated with the disease.

## 2. Patients and Methods

### 2.1. Patients

This study complied with the Declaration of Helsinki and was approved by the ethics committee of the Sister of Mercy University Hospital and the ethical committee of the University of Zagreb, School of Medicine (up-18-111). Informed consent was obtained from each patient prior to enrollment.

This case control cross sectional study included 126 patients with pituitary tumors: 60 non-functional pituitary tumor patients (30 males, average age 42 years, and 30 females, average age 48 years) and 66 functional pituitary tumor patients (20 males and 46 females, average age 42 years). Diagnosis was based upon their neuro-radiological findings and hormonal status, in comparison to the control group of 400 phenotypically healthy individuals from the Zagreb region (200 males and 200 females) who have never had any malignant disease. Differences of qualitative and quantitative traits of digito-palmar dermatoglyphics in patients with functional and non-functional pituitary tumors compared to the control group were analyzed separately in men and women since those two entities are acting entirely independently. 

### 2.2. Analysis of Dermatoglyphic Traits

The digito-palmar prints were taken and analyzed according to previously reported methods [9]. Hands were thoroughly washed with soap before taking prints. A requisite amount of ink was placed on the ink slab and an inverted “T”-shaped pad was soaked in it. The ink was evenly spread on the ink slab by light dusting. Afterwards, the fingers were rolled laterally on the ink slab, placed on white paper with one lateral edge, and then rolled over in the opposite direction. To take a palm print, the palm was lightly dusted with the same “T” pad. The palm was then kept on white paper with firm pressure applied on the center of the dorsum of hand and interdigital areas. Accordingly, dermatoglyphic patterns were recorded with a magnifying lens including qualitative parameters: fingertip patterns—arches, radial and ulnar loops, whorls, patterns in five interdigital areas-thenar/I1, I2, I3, I4, and hypothenar areas on the right and left hand); and quantitative parameters: finger ridge-counts on the right and left hand: FRR1, FRR2, FRR3, FRR4, FRR5, FRL1, FRL2, FRL3, FRL4, FRL5; palmar ridge counts (rc) on the right and on the left hand: a–b rc R, b–c rc R, c–d rc R; and a–b rc L, b–c rc L, c–d rc L, respectively, as well as the atd angles on both hands: atd R and atd L (dermatoglyphic trait formed by drawing lines between the triradii below the first and last digits and the most proximal triradius on the hypothenar region of the palm).

The method of counting was as follows: In a loop, a line was drawn from the core to the triradius and the ridges crossing the line were counted. The opening of the loop to ulnar or radial (side was noted as Lu or Lr). In a whorl, which has two triradii, counting was done with both triradii. From the core, a line was drawn to one triradius and in the same manner to the other triradius before counting was done.In an arch: the triradius is the core and hence the count is zero. The atd R and atd L angles: A line was drawn from axial triradius “t” to the digital triradii “a” and “d” and the angle was measured using a protractor. Variable a–b ridge count: the number of ridges crossing the line drawn from “a” to ‘b” was counted; b–c ridge count: the number of ridges crossing the line drawn from “b” to “c” was counted; c–d ridge count: the number of ridges crossing the line drawn from “c” to “d” was counted [9]. 

### 2.3. Statistical Methods

Quantitative parameters (finger ridge counts on the right and left hand: FRR1, FRR2, FRR3, FRR4, FRR5, FRL1, FRL2, FRL3, FRL4, FRL5; palmar ridge counts on the right and on the left hand: a–b rc R, b–c rc R, c–d rc R and a–b rc L, b–c rc L, c–d rc L, respectively, as well as the atd angles on both hands: atdR and atdL) were analyzed using ANOVA variance analysis. To determine the differences in quantitative traits between the different groups, ANOVA Tukey’s HDS *post hoc* test was used, which not only showed variance analysis between groups but also the actual difference in the arithmetical mean values and statistical significance—HSD (honestly significant difference) as well as equal distribution between variables. The level of significance was declared to be *p* < 0.05. The absolute and relative distribution of qualitative parameters (fingertip patterns-arches, radial and ulnar loops, whorls, and palmar patterns in five interdigital areas: thenar/I1, I2, I3, I4, and the hypothenar areas on the right and left hand) were analyzed using χ^2^ to determine the differences in the frequency of qualitative variables between healthy subjects and tumor patients. All analyses were performed using Statistical Package for Social Sciences software (SPSS PASW-Statistics 18).

## 3. Results

### 3.1. The Analysis of Quantitative Dermatoglyphic Traits

The results of ANOVA variance analysis comparing 18 quantitative variables of digito-palmar dermatoglyphics from pituitary tumor patients and control groups are presented in Table 1. In male subjects, the results have shown significant differences for the mean ridge count on the right little finger (FRR 5 *p* = 0.003) and all palmar ridge variables of both hands except for the b–c ridge count on the right hand between tumor patients and the control group. In female patients, a significant difference was observed for the mean ridge counts on both ring fingers (FRR 4 *p* = 0.003, FRL 4 *p* = 0.031) and the little finger of the right hand (FRR 5 *p* = 0.007), as well as for all palmar ridge variables of both hands, except for the b–c ridge count on the right hand, the same as in male patients.

The ANOVA Tukey’s HDS *post hoc* test enabled the identification of variables contributing to the heterogeneity among the investigated groups (statistically significant variables are presented in Table 2). Results have shown that, compared to the controls, males with functional pituitary tumors had a smaller ridge count for the a–b rc of both hands (*p* < 0.001), and the c–d rc R (*p* = 0.003), while those with non-functional tumors had a smaller ridge count for FRR 5 (*p* = 0.003), a–b rc, c–d rc of both hands and b–c rc L when compared to the control group. In female patients with functional pituitary tumors, differences in the ridge count for FRR 5, a–b rc and c–d rc of both hands and atd L were observed, whereas in the non-functional tumor group a difference in c–d rc R, FRR 4, and a–b rc of both hands was established (*p* = 0.003) compared to the control group (Table 2). When comparing functional with non-functional tumor groups, the biggest difference was observed in the mean ridges for c–d rc L in males (*p* = 0.023) and a–b rc R in females (*p* = 0.024). The variables that contribute the most to the heterogeneity among the investigated groups in both genders are the smaller ridge count between triradii a–b rc and c–d rc on the right hand. 

### 3.2. The Analysis of Qualitative Dermatoglyphic Traits 

Compared with the control group, the fingertip pattern of both hands in males showed a decrease in the total number of whorls (functional 20.0% *vs.* 33.9%; non-functional 26.7% *vs.* 33.9%) and slight increase in the total number of arches (functional 8.5% *vs.* 5.3%, non-functional 10.0% *vs.* 5.3%) and ulnar loops (functional 69.0% *vs.* 56.2%; non-functional 61.0% *vs.* 56.2%) (Table 3). Similar results for the fingertip patterns were also observed in female patients when compared to controls, except that the increase in the number of ulnar loops was limited to non-functional pituitary tumor patients (67.0% *vs.* 59.9%) (Table 3). 

The results of a χ^2^ test in male subjects have shown statistically significant differences in the fingertip patterns on the right hand (*p* < 0.001) and both hands together (*p* < 0.001) for functional tumor patients and on the left hand (*p* = 0.003) and both hands together (*p* < 0.001) for non-functional tumor patients when compared to controls. There were no significant differences between functional and non-functional tumor patients (Table 4). In female patients results were somewhat different. A significant difference was present for the right hand (*p* = 0.003) and both hands together (*p* < 0.001) between functional tumor group and controls, whereas the non-functional tumor group had different fingertip patterns on the right and left hand separately and both hands together (*p* < 0.001). When comparing functional with non-functional tumor groups, a difference was observed in the fingertip pattern of both hands together (*p* = 0.034) (Table 4). 

Palmar pattern distribution analysis in males (including hypothenar/I1 interdigital area, I2, I3, and I4 interdigital area, and thenar) revealed significant differences in the hypothenar when comparing functional tumor to non-functional tumor patients (*p* = 0.009) or controls (*p* = 0.014). Furthermore, non-functional tumor male patients had a different I4 interdigital area pattern (*p* = 0.016) than control group and a different thenar than functional tumor patients (*p* = 0.041) (statistically significant variables are presented in Table 5).

In female patients a significant difference was observed for the I2 and I3 interdigital area between non-functional tumor cases and control group (*p* = 0.003 and *p* = 0.021, respectively) and for the I3 interdigital area (*p* < 0.001) between the functional tumor and control groups (Table 5). 

## 4. Discussion

Presently there are no studies published on dermatoglyphic features in pituitary tumor patients. Therefore, the aim of this study was to determine an association between dermatoglyphic traits and pituitary adenomas, assuming there was a distinctive pattern of dermatoglyphics in patients with pituitary tumors. The results of the quantitative and qualitative analysis of digito-palmar dermatoglyphics in this study support the notion that patients with pituitary tumors can be distinguished as a separate biological group. Although we did not find markers that could specifically distinguish functional from non-functional tumors, we have found markers predisposing patients to the development of tumors in general (a small number of ridges between the triradius of both hands, a smaller number of ridges between triradius of c–d R), those for endocrine dysfunction (increased number of arches and reduced number of whorls, difference of pattern distribution in the I3 and I4 interdigital space), and some that could potentially be attributed to patients suffering from pituitary tumors (small number of ridges for variables FRR 5, smaller number of ridges in the FRL 4 of both hands, and a difference in the pattern distribution at the thenar of the I1 and I2 interdigital space). 

A number of inherited diseases caused by chromosomal aberrations (Down, Turner, and Klinefelter syndromes) correlate with characteristic dermatoglyphic patterns on the palms and feet, which makes them reliable indicators for chromosomal testing [9]. In the last two decades a large number of dermatoglyphic studies have been conducted in patients with malignant diseases: leukemia [17,18] and larynx [12], stomach [13], breast [11,14], colon [19], and lung [20] cancer, showing a correlation of certain dermatoglyphic properties with these diseases [21]. It is suggested that cancer patients could be distinguished from the general population by genes participating in the control of finger and palm dermatoglyphics development [22]. It is possible that these genes also predispose patients to the development of malignancy. All the abovementioned authors have found differences, especially in palmar dermatoglyphics, between the group of patients suffering from different types of carcinomas and healthy control groups. They have concluded that gene instability could be the basis for developing cancer later in life under the influence of environmental factors [11,12,13,14,17,18,19,20]. Therefore, differences in dermatoglyphics, apart from the possibility of pointing to a genetic/epigenetic cause of a disease, could also serve as a high-quality, easily available, and cheap marker for the discovery of a group with a predisposition for certain diseases. 

In general, our results are consistent with the results of previous studies and have confirmed our initial hypothesis. Significant differences in dermatoglyphic patterns in comparison to healthy people were found in both groups (patients with functional and non-functional tumors). The palmar variables making the greatest contribution to the heterogeneity among the investigated groups are, in both genders, a–b rc of both hands and c–d rc R. These results are in agreement with the results of previously mentioned studies of dermatoglyphic traits in malignant diseases [11,12,13,14,17,18,19,20], so these markers could be specific for all tumors in general.

It has been previously shown that qualitative traits are changing faster, while migration and microevolutionary factors affect the frequency of certain patterns in population groups. Those changes are perceived more quickly in men than in women. It is also considered that the qualitative properties in some cases are monogenetically determined (I2), but the influence of gender-related genes should not be ignored, since it could explain the gender difference in the number of ridges and in the size of the ATD angle [9,10]. The observed differences in the qualitative characteristics of dermatoglyphic traits confirm the established fact of their faster change during microevolution and their stronger susceptibility to genetic drift, but also a possible influence of environmental factors in early intrauterine development. Quantitative traits show the basic structure of a specific population and are inherited by a polygenic model, but they are less susceptible to change, genetic drift, and microevolutionary effects [9,10]. The observed differences in quantitative traits in our study could be a confirmation of a possible mutual genetic background to the development of pituitary tumors and dermatoglyphics, while in the etiology of the disease itself other (genetic/epigenetic) factors could have a more important role [8]. 

A comparison of male patients with functional and non-functional tumors, showed significant difference in the mean number of ridges for c–d rc L. Given that in previous research by Arrieta *et al.* [23] the number of ridges in the c–d rc region in men is affected mostly by heredity whereas the ridges in the a–b and b–c rc regions are influenced by environment, the results of our analysis confirm that a different genetic background causes functional and non-functional pituitary tumors. Since the comparison with the control group also demonstrated differences in other palmar variables (a–b rc, b–c rc, and c–d rc), we can assume that they are the result of the joint action of heredity and environmental factors on the development of dermatoglyphics and tumors.

A significant influence of heredity on the ridges in all three interdigital areas has been observed in females [23]; therefore, the differences found in this study (a smaller number of ridges in the a–b rc of both hands in all pituitary tumor patients, the c–d rc of both hands in patients with functional tumors, and the b–c rc L in patients with non-functional tumors) could be attributed to a possible genetic basis of dermatoglyphics or tumor development. However, besides those differences there were also changes in the FRR 4 and 5 and since it is known that the impact of heredity is somewhat smaller if we look at that particular parameter we can conclude that heredity and environment as well as their interaction are important in the formation of tumors [23,24].

In the qualitative traits analysis, our results regarding the number of whorls, radial loops, and arches in men and women with functional and non-functional pituitary tumors are consistent with previous findings implying that they too can be a general marker for the potential development of tumors, but not a specific marker for pituitary tumors [11,12,13,14,17,18,19,20]. However, some markers, such as a decrease in the total number of whorls, an increase in the total number of arches on both hands, and a difference in the palmar pattern distribution for the I3 and I4 interdigital areas could be specific to abnormal endocrine cell function, since they were also found in hypothyreosis and diabetes type 1 patients [25,26,27].

The present study has several limitations. The number of subjects was relatively small; therefore, larger scale studies are necessary to confirm our findings before applying dermatoglyphics as a diagnostic tool or a predictor of individual predisposition to development of pituitary tumors. Moreover, this study could potentially be criticized for the lack of comparative methods such as genetic analysis of specific genes potentially involved in pituitary tumor development. 

## 5. Conclusions

Our results have confirmed that there are differences in dermatoglyphic traits between healthy individuals and pituitary tumor patients as well as among two different groups of patients (functional *vs.* non-functional tumors), while at the same time identifying the features that make the greatest contribution to the abovementioned differences. Therefore, dermatoglyphic traits could serve as a potentially useful marker and a diagnostic tool, together with the usual methods, for identifying a specific group of individuals with a predisposition for acquiring pituitary adenoma, which would then lead to earlier detection and tumor treatment. 

## Figures and Tables

**Table 1:** Results of ANOVA variance analysis of quantitative dermatoglyphic traits between pituitary tumor patients (functional and non-functional) and controls.

Variable	Males		Females	
	F	*p < 0.05*	F	*p < 0.05*
*Right hand*				
FRR1	0.349	0.706	0.658	0.519
FRR2	0.158	0.854	1.672	0.190
FRR3	1.928	0.148	1.413	0.245
FRR4	2.258	0.107	5.845	* 0.003
FRR5	5.926	* 0.003	5.103	* 0.007
a–b rc R	16.648	* 0.000	31.573	* 0.001
b–c rc R	1.032	0.358	2.327	0.096
c–d rc R	8.923	* 0.000	11.064	* 0.001
atd R	3.378	* 0.036	4.335	* 0.014
*Left hand*				
FRL1	0.139	0.870	0.103	0.903
FRL2	1.336	0.265	0.143	0.867
FRL3	0.198	0.821	1.930	0.147
FRL4	2.547	0.080	3.522	* 0.031
FRL5	1.697	0.185	1.256	0.287
a–b rc L	21.277	* 0.000	26.045	* 0.001
b–c rc L	5.405	* 0.005	4.574	* 0.011
c–d rc L	7.666	* 0.001	7.460	* 0.001
atd L	7.914	* 0.000	8.487	* 0.001

* *p* < 0.05.

**Table 2:** ANOVA Tukey *post hoc* test analysis of quantitative dermatoglyphic traits in males and females comparing groups with functional tumors, non-functional tumors, and controls (statistically significant variables are presented).

Males						
	Functional *vs.* Non-Functional Tumors		Functional Tumors *vs.* Controls		Non-Functional Tumors *vs.* Controls	
	Diff. Mean	*p* < 0.05	Diff. Mean	*p* < 0.05	Diff. Mean	*p* < 0.05
FRR5	0.183	0.992	−2.685	0.068	−2.868	* 0.013
a–b rc R	1.317	0.776	−5.300	* 0.003	−6.617	* 0.001
b–c rc R	−0.400	0.967	−1.495	0.493	−1.095	0.579
c–d rc R	−1.167	0.796	−4.840	* 0.003	−3.673	* 0.009
atd R	1.583	0.767	−2.175	0.469	−3.758	0.041
a–b rc L	0.033	0.999	−6.980	* 0.001	−7.013	* 0.001
b–c rc L	2.300	0.392	−1.525	0.535	−3.825	* 0.004
c–d rc L	5.083	* 0.023	0.030	0.999	–5.053	* 0.001
atd L	2.767	0.386	–2.655	0.266	–5.422	* 0.001
Females						
FRR4	1.775	0.369	–1.671	0.164	–3.447	* 0.005
FRR5	–0.087	0.997	–2.287	* 0.022	–2.200	0.082
a–b rc R	–3.712	* 0.024	–7.508	* 0.001	–3.797	* 0.004
b–c rc R	0.914	0.817	–1.452	0.353	–2.367	0.147
c–d rc R	–1.304	0.662	–4.509	* 0.001	–3.205	* 0.030
atd R	0.809	0.899	–2.756	0.082	–3.565	0.054
FRL4	2.632	0.082	–0.060	0.997	–2.692	* 0.024
a–b rc L	–0.929	0.776	–6.016	* 0.001	–5.087	* 0.001
b–c rc L	1.033	0.757	–2.038	0.113	–3.072	* 0.031
c–d rc L	–0.877	0.848	–3.858	* 0.002	–2.982	0.067
atd L	1.454	0.699	–3.608	* 0.012	–5.062	* 0.003

* *p* < 0.05.

**Table 3:** Relative distribution of qualitative dermatoglyphic traits (fingertip patterns) in groups with tumors (functional and non-functional) and controls.

Gender	Group	Whorl %	Ulnar Loop %	Radial Loop %	Arch %
*Males*	Controls	33.9	56.2	4.5	5.3
	Non-functional tumors	26.7	61.0	2.3	10.0
	Functional tumors	20.0	69.0	2.5	8.5
*Females*	Controls	31.9	59.9	3.6	4.6
	Non-functional tumors	17.7	67.0	4.0	11.3
	Functional tumors	26.7	60.9	3.3	9.1

**Table 4:** Chi square test analysis of fingertip patterns (whorl, ulnar loop, radial loop, and arch) comparing groups with functional tumors, non-functional tumors and controls.

Fingertip Pattern	Functional *vs.* Non-Functional Tumors		Functional Tumors *vs.* Controls		Non-Functional Tumors *vs.* Controls	
**Males**						
	**χ^2^**	*p* < 0.05	**χ^2^**	*p* < 0.05	**χ^2^**	*p* < 0.05
Right hand	5.054	0.168	19.477	* 0.001	5.907	0.116
Left hand	2.894	0.408	6.494	0.090	14.276	* 0.003
Both hands	3.720	0.293	21.100	* 0.001	18.005	* 0.001
**Females**						
Right hand	7.079	0.069	25.472	* 0.003	13.686	* 0.001
Left hand	3.466	0.325	18.173	0.070	7.054	* 0.001
Both hands	8.692	* 0.034	41.002	* 0.001	17.048	* 0.001

* *p* < 0.05.

**Table 5:** Chi square test analysis of qualitative dermatoglyphic traits (palmar patterns) comparing groups with functional tumors, non-functional tumors and controls (statistically significant variables are presented).

Palmar Pattern	χ^2^	*p* < 0.05	Males
Hypothenar	6.096	0.014	Functional tumors *vs.* controls
I4 interdigital area	5.826	0.016	Non-functional tumors *vs.* controls
Hypothenar	6.871	0.009	Functional *vs.* non-functional tumors
Thenar	4.167	0.041	Functional *vs.* non-functional tumors
			**Females**
I3 interdigital area	22.186	0.001	Functional tumors *vs.* controls
I3 interdigital area	5.338	0.021	Non-functional tumors *vs.* controls
I2 interdigital area	8.887	0.003	Non-functional tumors *vs.* controls
